# Seasonal Sampling of a Microbial Community in the Sediment of Geoje-Hansan Bay, Republic of Korea

**DOI:** 10.1128/MRA.00566-21

**Published:** 2021-08-05

**Authors:** Seongsik Park, Hee-Eun Woo, Jong-Oh Kim, In-Cheol Lee, Seokjin Yoon, Kyunghoi Kim

**Affiliations:** a Department of Ocean Engineering, Pukyong National University, Busan, Republic of Korea; b Institute of Marine Biotechnology, Pukyong National University, Busan, Republic of Korea; c Dokdo Fisheries Research Center, National Institute of Fisheries Science, Pohang, Republic of Korea; University of Maryland School of Medicine

## Abstract

Several oyster farms are concentrated in Geoje-Hansan Bay, Republic of Korea, and there is concern about marine pollution. Hence, we monitored the sediment at this site for a year using 16S rRNA gene sequencing. The predominant phyla were *Proteobacteria* (69.9 to 79.1%) and *Bacteroidetes* (8.2 to 10.6%) in all seasons.

## ANNOUNCEMENT

Geoje-Hansan Bay, in the southeast part of South Korea, is a semienclosed bay with an area of 55 km^2^. Since aquaculture farms (especially oyster farms) are concentrated in this bay, there is a risk of marine pollution due to the accumulation of organic matter (1, 2). Marine sediment is an important repository of organic matter and nutrients that are deposited mainly from seawater and land (3). In particular, the microorganisms in sediment play an important role in the biogeochemical cycle of the ecosystem, including the decomposition of organic matter and the circulation of nutrients (4, 5). Nevertheless, monitoring of the marine pollution in Geoje-Hansan Bay is mainly focused on water quality management (6), and the sediment is managed mainly using organic matter (1). In the present study, the seasonal variation of the microbial community in the sediment of Geoje-Hansan Bay over a year was analyzed using 16S rRNA gene amplicon sequencing. The sediment was sampled using a grab sampler (Ponar Grab, 12.7 kg) at the western part of Geoje-Hansan Bay (34°50′45″N, 128°34′4″E) in April, August, October, and December 2019.

The temperature, pH, and oxidation reduction potential (ORP) of the collected sediment were measured with a pH/ORP meter (LAQUA D-53; HORIBA, Japan), and the results are shown in Table 1. Total DNA was extracted from 10 g of sediment using a DNeasy PowerMax soil kit (Qiagen), following the manufacturer’s protocol. According to a 16S metagenomic sequencing library preparation protocol (Illumina), a library was prepared using Herculase II Fusion DNA polymerase and the Nextera XT index kit v2. Quality control of the generated libraries was conducted using an Agilent 2100 Bioanalyzer. The library was sequenced on the Illumina MiSeq platform (300-bp paired-end format) at Macrogen, Inc. (Seoul, South Korea), and the numbers of raw reads are presented in Table 1. The adaptor sequences were removed from the raw reads using Cutadapt v1.11 (default settings) (7); then, the removed reads were merged using FLASH v1.2.11 (default settings) (8). The low-quality reads (Q < 20) were filtered out. The sequences were clustered by the number of operational taxonomic units (OTUs) using QIIME v1.8.0 (default settings) (9). Default parameters were used except where otherwise noted.

**TABLE 1:** Characteristics of sequencing data and collection site

Month	Temp (°C)	pH	ORP (mV)	No. of raw reads	No. of OTUs	SRA BioSample no.
April	18.1	7.5	−396.5	160,164	23,030	SRX10106073
August	26.1	7.4	−384.3	155,709	18,851	SRX10106074
October	20.2	7.6	−441.6	126,907	16,665	SRX10106075
December	7.8	7.8	−353.3	94,877	15,900	SRX10106076

**FIG 1:**
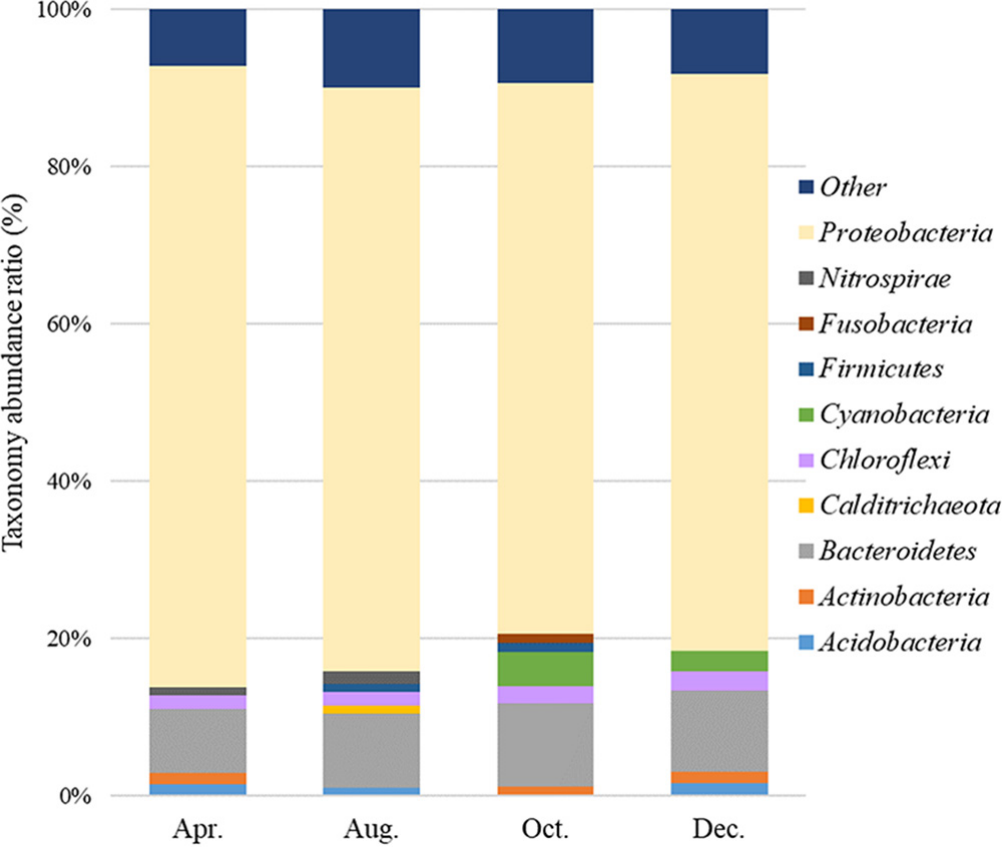
Ratio of the relative taxonomic abundance of the bacterial community in Geoje-Hansan Bay.

The predominant phyla were *Proteobacteria* (69.9 to 79.1%) and *Bacteroidetes* (8.2 to 10.6%) in all seasons (Fig. 1). *Chloroflexi* was the next most abundant phylum in April and August, followed by *Cyanobacteria* in October and December.

### Data availability.

The 16S rRNA gene amplicon sequences from this study have been deposited in the NCBI Sequence Read Archive (SRA) under accession number PRJNA701619.

## References

[1] Choi M, Lee I-S, Hwang D-W, Kim HC, Yoon S-P, Yun S, Kim C-S, Seo I-S. 2017. Organic enrichment and pollution in surface sediments from Jinhae and Geoje-Hansan bays with dense oyster farms. Fish Aquat Sci 50:777–787. doi:10.5657/KFAS.2017.0777.

[2] Cho Y, Lee W-C, Hong S, Kim H-C, Kim JB. 2012. GIS-based suitable site selection using habitat suitability index for oyster farms in Geoje-Hansan Bay, Korea. Ocean Coast Manag 56:10–16. doi:10.1016/j.ocecoaman.2011.10.009.

[3] Liu J, Yang H, Zhao M, Zhang X-H. 2014. Spatial distribution patterns of benthic microbial communities along the Pearl Estuary, China. Syst Appl Microbiol 37:578–589. doi:10.1016/j.syapm.2014.10.005. 25467555

[4] Oni OE, Schmidt F, Miyatake T, Kasten S, Witt M, Hinrichs K-U, Friedrich MW. 2015. Microbial communities and organic matter composition in surface and subsurface sediments of the Helgoland mud area, North Sea. Front Microbiol 6:1290. doi:10.3389/fmicb.2015.01290. 26635758 PMC4658423

[5] Cho H, Hwang CY, Kim J-G, Kang S, Knittel K, Choi A, Kim S-H, Rhee S-K, Yang EJ, Lee S, Hyun J-H. 2020. A unique benthic microbial community underlying the Phaeocystis antarctica-dominated Amundsen Sea polynya, Antarctica: a proxy for assessing the impact of global changes. Front Mar Sci 6:797. doi:10.3389/fmars.2019.00797.

[6] Kim D, Lee Y-J, Kang HY, Kwon K-Y, Lee W-C, Kwak JH. 2019. Seasonal variations in primary productivity and biomass of phytoplankton in Geoje-Hansan Bay on the southern coast of Korea. Ocean Sci J 54:213–227. doi:10.1007/s12601-019-0005-y.

[7] Martin M. 2011. Cutadapt removes adapter sequences from high-throughput sequencing reads. EMBnet J 17:10–12. doi:10.14806/ej.17.1.200.

[8] Magoč T, Salzberg SL. 2011. FLASH: fast length adjustment of short reads to improve genome assemblies. Bioinformatics 27:2957–2963. doi:10.1093/bioinformatics/btr507. 21903629 PMC3198573

[9] Kuczynski J, Stombaugh J, Walters WA, González A, Caporaso JG, Knight R. 2012. Using QIIME to analyze 16S rRNA gene sequences from microbial communities. Curr Protoc Microbiol 27:1E.5.1–1E.5.20. doi:10.1002/9780471729259.mc01e05s27. PMC4477843 23184592

